# Supplementary material for: Splice-Junction-Based Mapping of Alternative Isoforms in the Human Proteome
Source: Cell Rep. Author manuscript; Available in PMC 2020 Jan 15. (PMC6961840; doi:10.1016/j.celrep.2019.11.026)
Supplement: 3 [file NIHMS1546469-supplement-3.zip › DF2/PXD006675/TricuspidValve-110-Q9Y2S2-PNIQEAVEGAMHIQVNPPYYIPLVELVPHPETAPTTVDR.pdf]

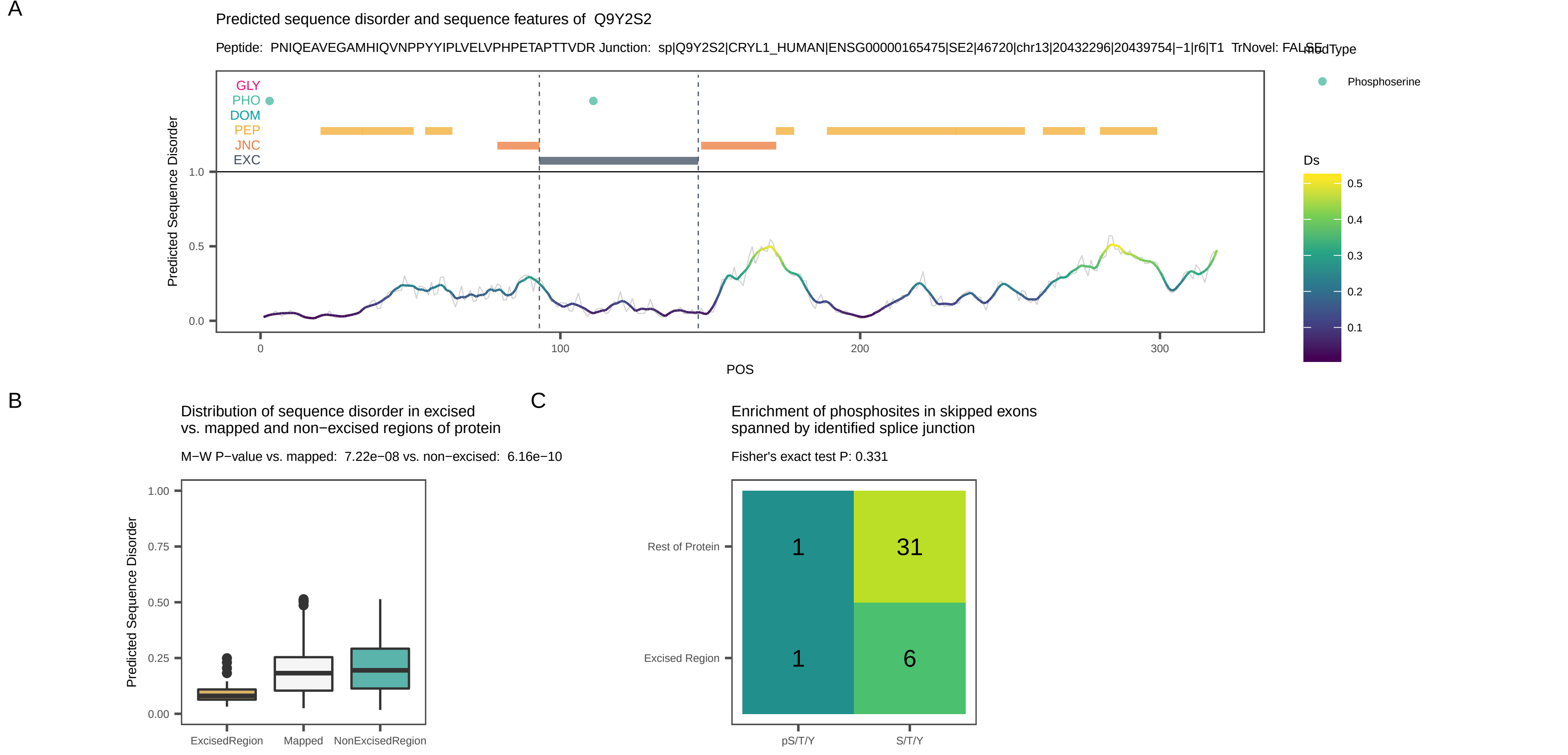
A
Predicted sequence disorder and sequence features of Q9Y2S2
Peptide: PNIQEAVEGAMHIQVNPPYYIPLVELVPHPETAPTTVDR Junction: sp|Q9Y2S2|CRYL1_HUMAN|ENSG00000165475|SE2|46720|chr13|20432296|20439754|−1|r6|T1 TrNovel: FALSE
modType
Phosphoserine
GLY
PHO
DOM
PEP
JNC
EXC
Ds
0.5
0.4
0.3
0.2
0.1
Predicted Sequence Disorder
1.0
0.5
0.0
0
100
200
300
POS
B
Distribution of sequence disorder in excised vs. mapped and non−excised regions of protein
M−W P−value vs. mapped: 7.22e−08 vs. non−excised: 6.16e−10
Predicted Sequence Disorder
1.00
0.75
0.50
0.25
0.00
ExcisedRegion
Mapped
NonExcisedRegion
C
Enrichment of phosphosites in skipped exons spanned by identified splice junction
Fisher's exact test P: 0.331
Rest of Protein
Excised Region
1
31
1
6
pS/T/Y
S/T/Y
